# 3D cardiac Chemical Shift Imaging of [1-13C] hyperpolarized acetate and pyruvate in pigs

**DOI:** 10.1186/1532-429X-15-S1-P10

**Published:** 2013-01-30

**Authors:** Luca Menichetti, Francesca Frijia, Alessandra Flori, Vincenzo Lionetti, Matteo Liserani, Giulio Giovannetti, Giacomo Bianchi, Simone L Romano, Vincenzo Positano, Jan Henrik Ardenkjaer-Larsen, Rolf F Schulte, Fabio A Recchia, Luigi Landini, Maria Filomena Santarelli, Massimo Lombardi

**Affiliations:** 1 Institute of Clinical Physiology, National Research Council, Pisa, Italy; 2 Fondazione G. Monasterio CNR-Regione Toscana, Pisa, Italy; 3 Scuola Superiore Sant'Anna, Pisa, Italy; 4 Faculty of Physics, University of Pisa, Pisa, Italy; 5 GE Healthcare, Hillerod, Denmark; 6 Department of Electrical Engineering, Technical University of Denmark, Kongens Lyngby, Denmark; 7 GE Global Research, Munich, Germany; 8 Department of Information Engineering, University of Pisa, Pisa, Italy

## Background

13C Dynamic Nuclear Polarization (DNP) with rapid dissolution together with Magnetic Resonance Chemical Shift Imaging (CSI) have been used for non-invasive real-time metabolic assessment in cardiac experimental models on a clinical 3T scanner. Here, we report an in vivo comparison of hyperpolarized [1-13C] pyruvate and [1-13C] acetate perfusion and metabolism: a method based on a 3D Spiral CSI sequence is presented for obtaining spatially and spectrally-resolved information on whole heart cardiac metabolism.

## Methods

In this work hyperpolarized [1-13C] pyruvate and [1-13C] acetate were injected in vivo to obtain spatially and spectrally resolved information of basal metabolism on whole heart in middle size animal models. Five healthy male farm pigs (38±2 kg) were studied in basal condition and subjected to imaging experiments performed on a 3T GE Signa HDx scanner using a 13C-quadrature birdcage coil (Rapid Biomedical). An HyperSense DNP polarizer (Oxford Inst.) was employed for the studies: a procedure for the hyperpolarization and dissolution of a large dose of TRIS-[1-13C]acetate water/glycerol mixture was set up while the preparation of a large dose of [1-13C] pyruvic acid was performed as recently published by this group. An anatomical region of interest covering the whole heart was first acquired with a proton reference scan and the metabolic information was then obtained using 3D IDEAL spiral CSI on the same region. Image re-slicing along cardiac short axis (SA) views and image fusion of 13C metabolite maps and anatomical 1H reference images were performed by PMOD software.

## Results

**Figure 1:**
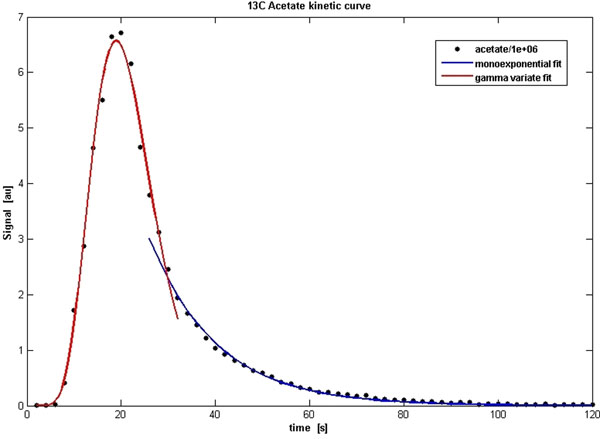
13C dynamic spectra were acquired using a slice selective pulse-and-acquire sequence (bandwidth 5000 Hz, 2048 pts, 10° FA). A long-axis slice of 20 mm was selected during excitation. Spectra were acquired from the beginning of the injection of the hyperpolarized [1-13C] acetate, every 2 s, for 120 s. Diagrammatic representation of the γ-variate and mono-exponential fitting of cardiac spectroscopic signal to obtain rate constants (N=4).

**Figure 2:**
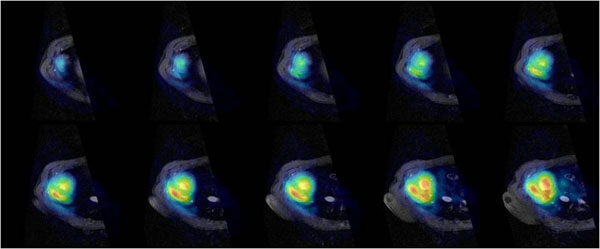
Representative maps in SA view of the heart showing the in vivo spatial distribution of hyperpolarized TRIS-[1-13C] acetate in pigs; spectroscopic data were normalized to the maximum value of signal amplitude.

A graph of the γ-variate and mono-exponential fitting of hyperpolarized [1-13C] acetate myocardial spectroscopic signals is reported in Figure 1 while a representative map in SA orientation through the heart is shown in Figure 2: [1-13C] acetate is extracted inside the heart and clearly detected in the heart-chambers and myocardial wall. Representative maps of spatial distribution of [1-13C]bicarbonate, [1-13C] lactate and [1-13C] pyruvate in SA orientation through the heart are also produced using hyperpolarized [1-13C] pyruvate.

## Conclusions

A comparison between acetate and pyruvate 13C-mapping has been realised as far as we know for the first time in pigs with this experimental approach. This ongoing study demonstrates the feasibility of whole-heart 13C-cardiac metabolic imaging in pigs for detecting and mapping cardiac metabolism in basal condition with hyperpolarized [1-13C]acetate in comparison with [1-13C] pyruvate.

This study is the first step towards the optimization of the [1-13C] acetate concentration and the acquisition sequence parameters to ensure suitable MR signals in myocardial tissue and to study its metabolic fate.

## Funding

Self funding.

